# Dietary patterns and nutritional habits of female students at Sulaimani university: a cross-sectional study in Sulaymaniyah, 2025–2026

**DOI:** 10.1186/s12889-025-25339-8

**Published:** 2025-11-18

**Authors:** Cheeman Salih Kakabra

**Affiliations:** Department of Community Health Nursing, College of Nursing, University of Sulaimani, Sulaimani, Kurdistan Region Iraq

**Keywords:** Dietary habits, Nutritional behavior, University students, Socioeconomic factors, Female health, Iraq

## Abstract

**Background:**

In Sulaymaniyah, Iraq, university students are increasingly exposed to dietary challenges influenced by socioeconomic, academic, and behavioral factors. This study aimed to assess the dietary patterns and nutritional habits of female students at the University of Sulaimani.

**Method:**

This cross-sectional study was conducted from January 15th, 2024, to September 20th, 2025, among female students enrolled in six colleges at the University of Sulaimani, using convenience sampling. The questionnaire included sociodemographic variables, a 14-item dietary habits scale, and a 23-item food frequency scale. Statistical analysis was performed using SPSS version 26 (IBM Corp., Armonk, NY). Chi-square tests assessed associations between dietary adequacy and demographic factors. Pearson correlation and multiple linear regression analyses were conducted to identify significant predictors of dietary behavior.

**Results:**

A total of 200 female students participated in the study. The mean dietary habit score was 29.4 ± 3.18, indicating overall moderate eating habits. Significant associations were identified between dietary adequacy and age, academic stage, father’s education, and perceived economic status (*p* < 0.01). Multiple linear regression analysis revealed that age, academic stage, father’s education, and economic status were significant positive predictors, whereas limited budget for healthy food negatively affected dietary adequacy. Strong positive correlations were also found between healthy food intake and overall eating habits (*r* = 0.72, *p* < 0.001).

**Conclusions:**

The study found that the participants had moderately eating habits overall, with only a minority demonstrating adequate dietary patterns. Therefore, it is recommended that policymakers and healthcare providers implement targeted interventions to enhance nutritional awareness, improve access to healthy food options, and support health literacy among university students.

## Introduction

Unhealthy dietary patterns are a major public health concern, the scope and long-term impacts of which have historically been underreported and undervalued. Rapid urbanization, shifting cultural norms, and growing exposure to processed foods have contributed to variable estimates of 30% to 70% prevalence of unhealthy eating behaviors among university students globally [1, 2]. In Iraq, recent national nutrition surveys revealed that approximately 42.3% of university-aged youth do not meet recommended daily nutrient intake levels, with notable disparities between genders. In Sulaymaniyah specifically, over 35% of female university students report relying on convenience foods more than five times per week. Although poor nutrition affects students worldwide, it is female students in low-resource settings, where food environments are often unsupportive, who are most at risk. Global calls for improving adolescent and youth nutrition were first initiated by the WHO in 2001, emphasizing the role of schools and universities in promoting dietary change [3]. However, comprehensive national strategies targeting university-level dietary behaviors have only recently begun to emerge, with the launch of the WHO Global Action Plan on Adolescent Health 2021–30 [4–6]. Unsurprisingly, poor dietary practices among university students have earned recognition as a “hidden epidemic” within noncommunicable disease prevention [7]. This is particularly true in middle-income countries like Iraq, where more than 60% of deaths are now attributed to NCDs linked to preventable lifestyle factors, including poor diet.

Unlike general population groups, university students are uniquely vulnerable to dietary transitions due to academic pressure, financial constraints, and exposure to new social environments. Those who are relatively health-conscious may still adopt irregular eating habits, such as meal skipping, late-night snacking, or high consumption of fast foods and sugary beverages [8]. In a regional study involving 1,200 Iraqi students, 64% of female respondents admitted to regularly skipping breakfast, while 48% reported consuming fast food at least three times weekly. In contrast, others face more systemic barriers including limited access to affordable healthy foods, inadequate nutrition education, or gender-based norms affecting food choices and body image. Female students, particularly in Middle Eastern contexts, often experience additional challenges such as familial expectations, dieting pressures, and cultural food restrictions, all of which may contribute to disordered eating behaviors [9–12]. Body image dissatisfaction has been identified in up to 70% of university females in recent Kurdish studies, which is strongly associated with restrictive eating and use of slimming products. Research from Iraq and neighboring countries indicates that over 50% of female university students may follow unhealthy diets characterized by low fruit and vegetable intake, high carbohydrate consumption, and inconsistent meal timing [13]. Moreover, iron-deficiency anemia among female students remains a key concern, with prevalence rates ranging between 22% and 38% in various Kurdish university settings.

Recommended dietary guidelines emphasize balanced intake across major food groups and consistency in eating behaviors, yet these are often unmet by students in university settings [1]. For instance, WHO suggests a daily intake of at least 400 g of fruits and vegetables; however, only 14% of female students in a 2023 Sulaymaniyah campus survey reported meeting this benchmark. While nutrition campaigns have been introduced in many regions, the effectiveness of such initiatives remains limited when food environments on campus are dominated by high-calorie, nutrient-poor options [14]. Many university cafeterias in Kurdistan offer few fresh or whole food choices, instead promoting inexpensive, processed snacks and sugary beverages. Despite the recognized role of nutrition in academic performance, physical health, and mental wellbeing, dietary interventions are rarely tailored to the daily routines and lived experiences of university students [1, 15–19]. In the Kurdistan Region, dietary trends among young adults are rapidly shifting toward Westernized patterns, influenced by media, socioeconomic changes, and the growth of fast-food chains [16, 20]. These shifts often go unmonitored, and with no localized dietary surveillance system in place, understanding students’ actual eating behaviors remains difficult. Furthermore, existing nutritional studies rarely disaggregate data by sex, thereby overlooking the specific vulnerabilities and needs of female students [1, 21]. However, there is a lack of localized evidence in the Kurdistan Region exploring how socioeconomic and academic factors jointly influence female university students’ dietary behaviors. This lack of disaggregated data represents a major blind spot in designing gender-sensitive nutritional policies and interventions.

Given the rising burden of noncommunicable diseases and the growing importance of early preventive behaviors, research on female student nutrition has become increasingly urgent. Sulaimani University, the largest public university in the region, hosts thousands of female students from diverse socioeconomic backgrounds and urban–rural settings [22]. Recent studies conducted in similar academic institutions have found that over 60% of female students do not meet national dietary recommendations for calcium, fiber, and iron [22–24]. These patterns raise concerns about long-term health risks including anemia, fatigue, poor academic concentration, and increased susceptibility to chronic disease. Therefore, this cross-sectional study aims to assess dietary patterns and nutritional habits among female students at Sulaimani University during the academic year 2025–2026.

### Research question

What are the dietary patterns and nutritional habits of female students at Sulaimani University?

## Methods

### Study design, setting, period, and sampling

This study was a cross-sectional study conducted in Sulaymaniyah, Iraq, involving female students enrolled at the University of Sulaimani. A convenience sampling method was used to collect data from the 15th of January 2024 to the 20th of September 2025. The selected colleges included Nursing College, Medicine College, Pharmacy College, Dentistry College, Islamic Education College, and Commercial College.

### Sample size

The sample size for this study was determined based on the total number of female students across the selected colleges of the University of Sulaimani. During the 9-month data collection period, a total of 200 female students who provided informed consent were included in the study, taking into account the overall female student population in the targeted colleges.

### Inclusion/exclusion

The inclusion criteria for participants included female students enrolled at the University of Sulaimani during the 2023–2025 academic years. Eligible participants were from any of the following colleges: Nursing, Medicine, Pharmacy, Dentistry, Islamic Education, and Commercial. Students who were unwilling to participate in the study or did not complete the questionnaire were excluded.

### Study tools and data collection

The questionnaire was divided into two main parts. The first part gathered demographic data, including age, marital status, college name, academic stage, parents’ educational levels, family history of chronic disease, and place of residence. The second part was a structured questionnaire designed to assess dietary habits and types of eating. For dietary habits, 14 multiple-choice statements were used to evaluate both healthy and unhealthy behaviors. Each item was rated on a 3-point scale: ‘Usually’ = 3, ‘Sometimes’ = 2, and ‘Never’ = 1. For items representing unhealthy behaviors, the scoring was reversed: ‘Usually’ = 1, ‘Sometimes’ = 2, and ‘Never’ = 3. For the food consumption section, a total of 23 items related to healthy and unhealthy food groups were rated using the same 3-point scale. Again, items reflecting unhealthy consumption were reverse-scored to ensure interpretive consistency. The questionnaire was provided in English, as the students’ educational system was conducted in the English language, and any unclear items were clarified by the researchers. Data were collected by distributing questionnaires to participants who met the inclusion criteria. Each participant was allotted a total of 10–15 minutes to complete the questionnaire.

### Pilot study

The study questionnaire, which assessed dietary habits and food consumption patterns, was initially tested with a group of 25 female students from the University of Sulaimani. The pilot study was conducted in September 2023 to evaluate the clarity, internal consistency, and relevance of the questionnaire items before the full-scale study. The internal consistency of the items was calculated using Cronbach’s alpha [25]. The overall Cronbach’s alpha was calculated as 0.81, indicating an acceptable level of internal consistency and reliability. It is important to note that the data from this initial pilot study were excluded from the final analysis.

### Measures

#### Sociodemographic characteristics

The first section of the questionnaire included sociodemographic information of the participants, such as age, marital status, college, academic stage, family size, perceived economic status, father’s and mother’s education levels, and family history of chronic diseases.

#### Dietary habits and food consumption questionnaire

To assess dietary patterns among female students at the University of Sulaimani, we used a structured dietary habits questionnaire. The dietary habits scale consisted of 14 items, each rated on a 3-point scale. Healthy behaviors such as eating meals at home, enjoying food variety, and regular meal patterns were positively scored (Usually = 3, Never = 1). For unhealthy behaviors such as eating at restaurants or consuming salty/fatty foods, reverse scoring was used (Usually = 1, Never = 3). The food consumption scale consisted of 23 items measuring the frequency of intake for healthy foods (e.g., vegetables, dairy, fruits, lean proteins) and unhealthy foods (e.g., fast food, processed items). Each item was rated as: Usually = 3, Sometimes = 2, and Never = 1, with reverse scoring applied for unhealthy food items. This scoring system allowed for the generation of overall dietary adequacy scores. The reliability of the questionnaire was confirmed through pilot testing, with Cronbach’s alpha calculated as 0.81 [25].

### Ethical approval and inform consent

This study followed the Institutional Research Ethics Board and the Declaration of Helsinki guidelines. Ethical approval for the study was obtained from the Scientific Committee at the College of Nursing, University of Sulaimani, on 17th March 2024, under approval number 6. Informed consent was obtained orally from all participants before they filled out the questionnaire. Anonymity and confidentiality of the participants were strictly maintained throughout the study.

###  Statistical analysis

Data were summarized and reported with frequency and percentage for categorical variables. Quantitative variables were presented with means and standard deviations. Chi-square tests were used to assess the associations between levels of dietary habits and sociodemographic variables. Pearson correlation analysis was conducted to examine the relationships between dietary behavior scores, food consumption patterns, and demographic factors. Multiple linear regression analysis was performed to identify significant predictors of dietary adequacy scores. All statistical analyses were conducted using SPSS version 26 (IBM Corp., Armonk, NY), and significance was considered at *p* < 0.05.

## Results

### Demographic characteristics

A total of 200 female students from Sulaimani University participated in this cross-sectional study. The mean age was 20.7 ± 1.69 years, with most participants aged 20–21 years (38.5%). The majority were enrolled in the Nursing (42.0%) and Medicine (39.5%) colleges, while smaller proportions attended Dentistry, Islamic, Commercial, and Pharmacy colleges. Most students were in the 2nd (25.5%) and 4th (25.0%) academic stages and were single (93.0%). Nearly three-quarters (73.0%) reported having more than five family members. In terms of economic status, 54.0% perceived their income as sufficient, and 45.5% as barely sufficient. Over half (52.5%) used the internet for dietary advice, and 45.0% reported a limited budget for healthy food. Fathers’ education was mainly primary (36.5%) or non-formal (17.5%), while mothers’ education levels were similar, with 38.0% having completed primary education and 31.0% non-formal. Overall, eating habits were categorized as inadequate (38.0%), moderate (32.0%), and adequate (30.0%), with a mean score of 29.4 ± 3.18, indicating moderate dietary behavior among participants. Detailed demographics and other variables are presented in Table 1.

**Table 1:** Demographic characteristics of participants

No.	Variables	Characteristics *n* = 200	F	%
1.	Age (year)	18–19 years	40	20.00
		19–20 years	60	30.00
		20–21 years	77	38.50
		> 22 years	23	11.50
		Mean ± SD	20.70 ± 1.69	
2.	College	Medicine	79	39.50
		Pharmacy	8	4.00
		Islamic	9	4.50
		Nursing	84	42.00
		Commercial	5	2.50
		Dentistry	15	7.50
3.	Stage	1 st stage	41	20.50
		2nd stage	51	25.50
		3rd stage	44	22.00
		4th stage	50	25.00
		5th stage	13	6.50
		6th stage	1	0.50
4.	Marital Status	Married	8	4.00
		Single	186	93.00
		Other	6	3.00
5.	Number of Family Members	< 5 members	54	27.00
		> 5 members	146	73.00
6.	Perceived Economic Status	Sufficient	108	54.00
		Barely sufficient	91	45.50
		Insufficient	1	0.50
7.	Use Internet for Dietary Advice	Yes	105	52.50
		No	95	47.50
8.	Limited Budget for Healthy Foods	Yes	90	45.00
		No	110	55.00
9.	Father’s Education Level	Non-formal education	35	17.50
		Primary	73	36.50
		Preparatory	38	19.00
		Institute/College	21	10.50
		Postgraduate	33	16.50
10.	Mother’s Education Level	Non-formal education	62	31.00
		Primary	76	38.00
		Preparatory	28	14.00
		Institute/College	15	7.50
		Postgraduate	19	9.50
11.	Eating Habit Levels	Inadequate eating habits	76	38.0
		Moderately eating habits	64	32.0
		Adequate eating habits	60	30.0
		Mean ± SD	29.4 ± 3.18	

*F* Frequency, %= Percentage

### Health status and family medical history

The results showed that the majority of students (93.0%) reported having no chronic health conditions, while only 7.0% had a diagnosed illness. Regarding family medical history, 95.0% indicated no known hereditary diseases, with small proportions reporting cardiovascular disease (1.5%), diabetes mellitus (0.5%), hypertension (0.5%), or other conditions such as respiratory or autoimmune disorders (2.5%). For paternal medical history, 64.5% reported no known conditions, whereas diabetes mellitus (18.0%) and cardiovascular disease (10.0%) were the most frequent. Similarly, among maternal histories, 63.5% reported no illness, with hypertension (17.0%), diabetes mellitus (6.0%), and cardiovascular disease (5.0%) being the most common. For further details, see Table 2.

**Table 2:** Health status and family medical history of female students at Sulaimani university (*n* = 200)

No.	Variables	Categories	F	%
1	Student’s Chronic Health Condition	Has a chronic disease	14	7.00
		No chronic disease	186	93.00
2	Personal Family Disease History (From student’s side)	No known family disease	190	95.00
		Cardiovascular disease	3	1.50
		Diabetes mellitus	1	0.50
		Hypertension	1	0.50
		Other conditions*	5	2.50
3	Father’s Medical History	No known disease	129	64.50
		Cardiovascular disease	20	10.00
		Diabetes mellitus	36	18.00
		Hypertension	4	2.00
		Cancer	0	0.00
		Other conditions*	11	5.50
4	Mother’s Medical History	No known disease	127	63.50
		Cardiovascular disease	10	5.00
		Diabetes mellitus	12	6.00
		Hypertension	34	17.00
		Cancer	5	2.50
		Other conditions*	12	6.00

“Others” in family disease history includes respiratory, gastrointestinal, and autoimmune conditions. “None” indicates the absence of any known diagnosis in that category

### Dietary behaviors of female students

The results revealed that the majority of students (87.0%) reported always eating at home, with a high mean score of 2.86, indicating a strong preference for home meals. In contrast, 76.0% never ate at relatives’ houses, and 63.5% only sometimes ate at restaurants, reflecting a moderate tendency for outside eating (mean score = 2.72). Over half of the students (57.0%) always ate meals regularly, yet patterns such as eating fruit before sleep (23.0% always) and eating food at any time (43.5% always) suggest inconsistency in timing and control. Notably, 68.5% always enjoyed a wide variety of foods, reflecting dietary diversity. However, 42.0% often felt hungry, and 32.5% reported always finishing meals quickly, implying possible issues with meal sufficiency or satiety. Behavioral cues were strong, as 64.0% always felt an urge to eat upon seeing or smelling favorite foods. Other less healthy habits were also reported, such as 43.5% always drinking tea/coffee right after meals, and frequent intake of salty (35.0%) and fatty foods (29.5%). Lastly, 18.5% always swallowed food without proper chewing, potentially affecting digestion and satiety. For more details, refer to Table 3.

**Table 3:** Dietary behaviors of female students at Sulaimani university (*n* = 200)

No.	Items	Always (F/%)	Sometimes (F/%)	Never (F/%)	Mean Score
1.	Eating at home	174 (87.0%)	25 (12.5%)	1 (0.5%)	2.86
2.	Eating at relatives’ houses*	20 (10.0%)	28 (14.0%)	152 (76.0%)	2.66
3.	Eating at restaurants*	43 (21.5%)	127 (63.5%)	30 (15.0%)	2.72
4.	Eating meals regularly	114 (57.0%)	67 (32.5%)	21 (10.5%)	2.46
5.	Eating fruit before sleep*	46 (23.0%)	97 (48.5%)	57 (28.5%)	2.05
6.	Eating food at any time*	87 (43.5%)	85 (42.5%)	28 (14.0%)	1.70
7.	Enjoying a wide variety of foods	137 (68.5%)	51 (25.5%)	12 (6.0%)	2.62
8.	Often feeling hungry*	84 (42.0%)	97 (48.5%)	19 (9.5%)	1.67
9.	Often finishing meals quickly*	65 (32.5%)	76 (38.0%)	59 (29.5%)	1.97
10.	Feeling the urge to eat when seeing/smelling favorite food*	128 (64.0%)	59 (29.5%)	13 (6.5%)	1.42
11.	Drinking tea/coffee directly after meals*	87 (43.5%)	51 (25.5%)	62 (31.0%)	1.88
12.	Eating foods high in salt (e.g., salted nuts)*	70 (35.0%)	77 (38.5%)	53 (26.5%)	1.92
13.	Eating fatty/oily foods (e.g., butter)*	59 (29.5%)	93 (46.5%)	48 (24.0%)	1.95
14.	Swallowing food without enough chewing (mastication)*	37 (18.5%)	56 (28.0%)	107 (53.5%)	2.35

Items marked with an asterisk relate to culturally specific or behavioral patterns such as eating at relatives’ homes or consuming salted/fatty foods.*. F = Frequency; % = Percentage

### Consumption patterns across food categories

The results revealed that the most frequently consumed items were herbs and seasoning (87.5%), white bread (87.0%), and nuts and seeds (71.5%), with mean scores of 2.84, 1.17, and 2.62, respectively. High intake of processed foods such as fast food (74.5%) and high-fat spreads (61.0%) was also noted. In contrast, healthier options like fresh vegetables (64.0%), boiled vegetables (57.5%), and legumes (41.0%) showed good but moderate consumption. Lean protein sources such as chicken (50.5%) were preferred over red meat (25.5%). Overall, the dietary profile reflects a mix of nutritious habits alongside frequent consumption of high-fat, salty, and processed foods. For extra details, refer to Table 4.

**Table 4:** Frequency of consumption of various food categories among female students at Sulaimani university (*n* = 200)

No.	Food Category	Always (F/%)	Sometimes (F/%)	Never (F/%)	Mean Score
1.	Lean red meat	51 (25.5%)	109 (54.5%)	40 (20.0%)	2.05
2.	Drinking tea or coffee	32 (16.0%)	104 (52.0%)	64 (32.0%)	2.16
3.	Chicken or poultry	101 (50.5%)	78 (39.0%)	21 (10.5%)	2.40
4.	Egg	78 (39.0%)	53 (26.5%)	69 (34.5%)	2.04
5.	White bread (processed)*	174 (87.0%)	17 (8.5%)	9 (4.5%)	1.17
6.	White rice (processed)*	100 (50.0%)	63 (31.5%)	37 (18.5%)	1.68
7.	Whole grain oat	109 (54.5%)	73 (36.5%)	18 (9.0%)	2.45
8.	Dairy products	128 (64.0%)	50 (25.0%)	22 (11.0%)	2.53
9.	Fried food*	65 (32.5%)	102 (51.0%)	33 (16.5%)	1.84
10.	Fast food (e.g., pizza, burger)*	148 (74.5%)	31 (15.5%)	20 (10.0%)	1.35
11.	Boiled vegetables (e.g., broccoli, zucchini)	115 (57.5%)	65 (32.5%)	20 (10.0%)	2.47
12.	Fresh vegetables (e.g., cucumber)	128 (64.0%)	40 (20.0%)	32 (16.0%)	2.48
13.	Fizzy drinks*	113 (56.5%)	74 (37.0%)	13 (6.5%)	1.50
14.	Desserts (e.g., cake, biscuits, jam, candy, jelly)*	73 (36.5%)	82 (41.0%)	45 (22.5%)	1.86
15.	Fruit	86 (43.0%)	94 (47.0%)	20 (10.0%)	2.33
16.	Legumes (e.g., beans, chickpeas)	82 (41.0%)	96 (48.0%)	22 (11.0%)	2.30
17.	Nuts and seeds	143 (71.5%)	39 (19.5%)	18 (9.0%)	2.62
18.	High-fat spreads (e.g., food cream, margarine)*	122 (61.0%)	52 (26.0%)	26 (13.0%)	1.52
19.	Herbs and seasoning	175 (87.5%)	18 (9.0%)	7 (3.5%)	2.84
20.	Canned foods*	63 (31.5%)	92 (46.0%)	45 (22.5%)	1.91
21.	Ketchup and mayonnaise*	79 (39.5%)	81 (40.5%)	40 (20.0%)	1.80
22.	Prepared noodles and pasta*	123 (61.5%)	54 (27.0%)	23 (11.5%)	2.50

Items marked with an asterisk relate to culturally specific or behavioral patterns such as eating at relatives’ homes or consuming salted/fatty foods.*. F = Frequency; % = Percentage

### Correlation between legal knowledge, good attitude, interference of nurses to legal issues, and demographic variables

The analysis revealed several significant associations between dietary habit levels and demographic characteristics. Students aged over 22 years were more likely to demonstrate adequate dietary habits (38.3%), compared to those aged 18–22 years, among whom 98.7% had inadequate habits (*p* < 0.01). Across academic colleges, none of the students reported fully adequate dietary patterns, though Nursing students (37.5%) had the highest proportion of moderate habits (*p* < 0.01). Academic stage showed a strong relationship with dietary adequacy, as all first-stage students exhibited inadequate habits, while 92.0% of fourth-stage and 100% of fifth- and sixth-stage students demonstrated adequate patterns (*p* < 0.01). Father’s education level was also a significant factor; all students whose fathers held college or postgraduate degrees exhibited adequate dietary habits, whereas none of those with non-formally educated fathers showed even moderate habits (*p* < 0.01). Economic status further influenced outcomes—65.6% of students with barely sufficient income reported adequate habits, compared to none among those with sufficient income. For further details, refer to Table 5.

**Table 5:** Association between Socio-Demographic variables and levels of dietary habits among female students

No.	Socio-Demographic Variable	Category	Inadequate F (%)	Moderate F (%)	Adequate F (%)	Test Result	*p*-value
1.	Age (years)	18–22 years	75 (98.7%)	64 (100.0%)	37 (61.7%)	χ² = 12.7	< 0.01
		> 22 years	1 (1.3%)	0 (0.0%)	23 (38.3%)		
2.	College	Medicine	75 (98.7%)	4 (6.3%)	0 (0.0%)	χ² = 18.4	< 0.01
		Pharmacy	0 (0.0%)	8 (12.5%)	0 (0.0%)		
		Islamic	0 (0.0%)	9 (14.1%)	0 (0.0%)		
		Nursing	0 (0.0%)	24 (37.5%)	0 (0.0%)		
		Economy	0 (0.0%)	5 (7.8%)	0 (0.0%)		
		Dentistry	0 (0.0%)	15 (23.4%)	0 (0.0%)		
3.	Stage of Study	1 st stage	41 (100.0%)	0 (0.0%)	0 (0.0%)	χ² = 12.3	< 0.01
		2nd stage	35 (68.6%)	16 (31.4%)	0 (0.0%)		
		3rd stage	0 (0.0%)	44 (100.0%)	0 (0.0%)		
		4th stage	0 (0.0%)	4 (8.0%)	46 (92.0%)		
		5th stage	0 (0.0%)	0 (0.0%)	13 (100.0%)		
		6th stage	0 (0.0%)	0 (0.0%)	1 (100.0%)		
4.	Marital Status	Married	8 (100.0%)	0 (0.0%)	0 (0.0%)	χ² = 5.5	0.33
		Single	62 (33.3%)	64 (34.4%)	60 (32.3%)		
		Other	6 (100.0%)	0 (0.0%)	0 (0.0%)		
5.	Perceived Economic Status	Sufficient	76 (69.7%)	32 (30.3%)	0 (0.0%)	χ² = 5.8	< 0.01
		Barely sufficient	0 (0.0%)	32 (34.4%)	59 (65.6%)		
		Insufficient	0 (0.0%)	0 (0.0%)	1 (100.0%)		
6.	Father’s Education Level	Non-formal	35 (100.0%)	0 (0.0%)	0 (0.0%)	χ² = 182.4	< 0.01
		Primary	41 (56.2%)	32 (43.8%)	0 (0.0%)		
		Preparatory	0 (0.0%)	32 (84.2%)	6 (15.8%)		
		Institute/College	0 (0.0%)	0 (0.0%)	21 (100.0%)		
		Postgraduate	0 (0.0%)	0 (0.0%)	33 (100.0%)		

Dietary adequacy was categorized as Inadequate, Moderate, and Adequate. Chi-square tests were used to determine statistical significance, and Significance was set at *p* < 0.05

### Predictors of dietary adequacy score among female students

The results showed that age was a significant positive predictor of dietary adequacy (B = 0.23, *p* = 0.005), indicating that older students tended to have higher adequacy scores. Stage of study also had a strong positive effect (B = 0.34, *p* < 0.001), suggesting that students in higher academic stages were more likely to maintain adequate dietary habits. Among socioeconomic variables, perceived economic status (B = 0.52, *p* = 0.001) and father’s education level (B = 0.41, *p* < 0.001) were significant predictors, both associated with better dietary adequacy. In contrast, having a limited budget for healthy foods negatively predicted dietary adequacy (B = −0.67, *p* < 0.001). Additionally, using the internet for dietary advice was positively associated with higher scores (B = 0.43, *p* = 0.012). Notably, nursing students had significantly lower scores compared to medicine students (B = −0.45, *p* = 0.034), while other variables such as mother’s education and family medical history were not significant. For additional details, refer to Table 6.

**Table 6:** Multiple linear regression analysis for factors predicting dietary adequacy score among female students at Sulaimani university (*n* = 200)

Predictor Variable	B	SE	β	t	*p*-value	95% CI
Demographic Factors						
Age (years)	0.23	0.08	0.18	2.87	0.005**	[0.07, 0.39]
College (Ref: Medicine)						
Nursing	−0.45	0.21	−0.15	−2.14	0.034*	[−0.86, −0.04]
Pharmacy	−0.32	0.35	−0.06	−0.91	0.364	[−1.01, 0.37]
Others	−0.28	0.26	−0.08	−1.08	0.282	[−0.79, 0.23]
Study Stage	0.34	0.09	0.31	3.78	< 0.001***	[0.16, 0.52]
Socioeconomic Factors						
Perceived Economic Status	0.52	0.16	0.24	3.25	0.001**	[0.20, 0.84]
Father’s Education Level	0.41	0.11	0.28	3.73	< 0.001***	[0.19, 0.63]
Mother’s Education Level	0.18	0.12	0.12	1.50	0.135	[−0.06, 0.42]
Limited Budget for Healthy Foods	−0.67	0.18	−0.22	−3.72	< 0.001***	[−1.02, −0.32]
Health-Related Factors						
Family History of Chronic Disease	−0.29	0.31	−0.05	−0.94	0.349	[−0.90, 0.32]
Use Internet for Dietary Advice	0.43	0.17	0.16	2.53	0.012*	[0.09, 0.77]

Dietary Adequacy Score is the dependent variable, *B* Unstandardized Coefficient, *SE* Standard Error, *β* Standardized Coefficient, *CI* Confidence Interval. *p < 0.05, **p < 0.01, **p < 0.001

### Correlations between dietary Behaviors, food Consumption, and demographic variables

The results showed strong positive correlations between overall eating habits and both healthy food consumption (*r* = 0.72, *p* < 0.001) and regular meal patterns (*r* = 0.65, *p* < 0.001). Conversely, processed food consumption was negatively associated with overall eating habits (*r* = −0.58, *p* < 0.001) and healthy food intake (*r* = −0.64, *p* < 0.001). Fast food frequency showed a moderate positive correlation with processed food intake (*r* = 0.69, *p* < 0.001) but negative correlations with healthy behaviors. Notably, fruit and vegetable intake had strong positive relationships with healthy food score (*r* = 0.78) and overall eating habits (*r* = 0.59). Among demographic factors, age and economic status were positively associated with better eating patterns (*r* = 0.31 and *r* = 0.42, respectively), while larger family size was weakly but significantly associated with poorer eating habits (*r* = −0.16, *p* < 0.05). For more details, refer to Table 7.

**Table 7:** Correlation matrix between dietary behavior scores and food consumption patterns (*n* = 200)

No.	Variables	1.	2.	3.	4.	5.	6.	7.	8.	9.	10.
1.	Overall Eating Habits Score	1.00									
2.	Healthy Food Consumption Score	0.72***	1.00								
3.	Processed Food Consumption Score	−0.58***	−0.64***	1.00							
4.	Regular Meal Pattern Score	0.65***	0.54***	−0.41***	1.00						
5.	Fast Food Frequency	−0.48***	−0.52***	0.69***	−0.38***	1.00					
6.	Fruit & Vegetable Intake	0.59***	0.78***	−0.45***	0.43***	−0.39***	1.00				
7.	Dairy Products Consumption	0.44***	0.61***	−0.32***	0.36***	−0.28***	0.47***	1.00			
8.	Age	0.31***	0.28***	−0.22**	0.26***	−0.19**	0.24**	0.18*	1.00		
9.	Family Size	−0.16*	−0.14*	0.12	−0.11	0.15*	−0.13	−0.09	−0.08	1.00	
10.	Economic Status Score	0.42***	0.38***	−0.35***	0.33***	−0.31***	0.36***	0.29***	0.21**	−0.23**	1.00

Note: Values are Pearson correlation coefficients, and *p < 0.05, **p < 0.01, **p < 0.001

## Discussion

The present study was conducted to assess dietary patterns and nutritional habits among female students at Sulaimani University during the academic year 2025–2026. Overall, the results revealed that participants demonstrated moderate dietary behaviors, with considerable variation across demographic and socioeconomic factors. The majority of students exhibited a preference for home-cooked meals, yet patterns of processed food consumption and irregular eating behaviors were evident, suggesting a complex interplay between traditional dietary practices and modern lifestyle influences.

University students, particularly young women, represent a critical demographic for nutritional interventions, as dietary habits established during this period often persist into adulthood [26, 27]. In Sulaymaniyah, rapid urbanization and evolving food environments have created unique challenges for maintaining healthy dietary patterns among university students. Despite growing concerns about nutritional inadequacy among this population, there remains a significant gap in understanding the specific dietary behaviors and consumption patterns of female students in Iraqi Kurdistan. Research examining the interplay between socioeconomic factors, educational background, and dietary adequacy in this region is particularly scarce. Given the importance of these details, we aimed to comprehensively assess dietary patterns and nutritional habits among female students at Sulaimani University to inform targeted public health interventions.

The demographic profile of our study participants, predominantly young female students with a mean age of approximately twenty-one years and enrolled primarily in health sciences programs, provides valuable insights into the nutritional landscape of university education in Sulaymaniyah. The concentration of participants in nursing and medicine programs reflects the gender distribution commonly observed in health sciences education across Middle Eastern universities [28, 29]. The predominance of single students with large family sizes, where nearly three-quarters reported having more than five family members, aligns with typical family structures in Iraqi society and throughout the broader Kurdistan region. This demographic characteristic may influence dietary behaviors through shared family meals and traditional food preparation methods, which are deeply embedded in Kurdish cultural practices.

The health status data revealed that the overwhelming majority of students reported no chronic health conditions, suggesting a generally healthy young adult population. However, the family medical history patterns, particularly the notable prevalence of diabetes mellitus and cardiovascular disease among fathers, and hypertension among mothers, warrant attention as these conditions have strong dietary links and may increase students’ future health risks [30]. The relatively high reporting of parental chronic diseases, especially metabolic conditions, underscores the importance of preventive dietary interventions during the university years.

The dietary behaviors exhibited by participants revealed both encouraging and concerning patterns. The strong preference for home-based eating, with the vast majority reporting always eating at home, represents a protective factor that has been associated with better nutritional outcomes in numerous studies across diverse populations [31, 32]. Home food preparation typically allows for greater control over ingredients, portion sizes, and cooking methods, potentially reducing intake of excess sodium, unhealthy fats, and added sugars. However, this positive pattern was counterbalanced by concerning behaviors such as irregular meal timing, rapid eating, and significant consumption of tea or coffee immediately following meals, which may interfere with nutrient absorption, particularly iron.

The behavioral triggers observed, particularly the strong urge to eat upon encountering favorite foods through sight or smell, reflect the powerful influence of environmental and sensory cues on eating behavior. This phenomenon, well-documented in nutritional psychology literature, can lead to overconsumption and difficulty maintaining dietary control [31, 33, 34]. The reported pattern of frequent hunger and rapid meal completion may indicate inadequate portion sizes, poor satiety from food choices, or eating in response to environmental cues rather than physiological hunger. These behaviors are particularly common among young adults managing academic stress and time constraints, which often lead to compromised meal quality and eating patterns. Examining the specific foods consumed provides further insight into nutritional adequacy.

The moderate consumption of vegetables, legumes, and lean proteins suggests that while students maintain some awareness of healthy food choices, consistent adherence remains challenging. The preference for chicken over red meat aligns with both economic considerations and contemporary nutritional guidance emphasizing lean protein sources [35, 36]. However, the overall dietary profile, combining beneficial traditional elements with problematic modern additions, creates nutritional vulnerability. This pattern has been associated with increased risk for obesity, metabolic syndrome, and other diet-related chronic diseases in populations undergoing nutritional transition [37, 38]. Understanding which factors predict better or poorer dietary adequacy becomes crucial for developing targeted interventions.

The significant associations between dietary adequacy and various demographic factors revealed important intervention targets. The positive relationship between age and dietary adequacy, along with academic stage, suggests that maturation and educational progression contribute to improved nutritional awareness and behavior. This finding aligns with research demonstrating that older students and those in advanced academic stages typically exhibit greater health consciousness and better dietary practices [39, 40]. The powerful influence of paternal education on dietary adequacy, where students with highly educated fathers consistently demonstrated adequate habits, highlights the role of family educational background in shaping nutritional behaviors. Parental education has been extensively linked to offspring health behaviors through multiple pathways, including health literacy, resource allocation, and role modeling [31, 33].

The strong positive correlations between overall eating habits and both healthy food consumption and regular meal patterns emphasize the interconnected nature of dietary behaviors. Students who maintained structured eating schedules were more likely to consume nutritious foods and avoid excessive processed food intake, suggesting that temporal regularity in eating may support better food choices [41]. The inverse relationships between processed food consumption and healthy eating indicators highlight how these patterns tend to be mutually exclusive rather than coexisting. The particularly strong association between fruit and vegetable intake and overall diet quality reflects the well-established role of these foods as markers of healthy dietary patterns [26, 27]. The positive associations of age and economic status with better eating patterns, while family size showed weak negative associations, suggest that individual maturation and financial resources facilitate healthy eating, whereas larger households may strain resources or complicate meal planning in ways that compromise nutritional quality.

Despite the valuable insights provided by this study, several limitations should be acknowledged. The cross-sectional design limits the ability to infer causality between socioeconomic factors and dietary patterns, as temporal relationships cannot be established. Additionally, the study focused exclusively on female students at one university, which may limit the generalizability of findings to male students, other educational institutions, or non-university young adults in the region. The reliance on self-reported dietary data may introduce recall bias and social desirability bias, potentially affecting accuracy. Future research should employ longitudinal designs to track dietary pattern evolution throughout university education and assess the long-term health impacts of different eating behaviors. Intervention studies examining the effectiveness of nutritional education programs tailored to the specific needs and constraints of university students in this region would provide practical guidance for improving dietary adequacy. Additionally, comparative studies including male students and diverse educational programs would offer a more comprehensive understanding of nutritional challenges among Iraqi Kurdistan’s university population.

## Conclusion

The study revealed that participants generally exhibited moderate eating habits, while only a small proportion achieved adequate dietary patterns. Consequently, there is a need for policymakers and healthcare professionals to develop tailored strategies that promote nutritional education, facilitate healthier food choices, and strengthen dietary literacy among university students. Future research should explore the effectiveness of campus-based nutrition programs and assess long-term behavioral changes. Expanding such studies to include male students and multiple institutions could offer a more comprehensive view of dietary trends among the youth.

## Data Availability

The data that support the findings of this study are available from the corresponding author upon reasonable request.
